# A case of emmonsiosis in an HIV-infected child

**DOI:** 10.4102/sajhivmed.v16i1.352

**Published:** 2015-06-11

**Authors:** Harsha Lochan, Preneshni Naicker, Tsidiso Maphanga, Anthea Ryan, Komala Pillay, Nelesh P. Govender, Brian Eley

**Affiliations:** 1 Paediatric Infectious Diseases Unit, Red Cross War Memorial Children's Hospital, South Africa; 2 Department of Paediatrics and Child Health, University of Cape Town, South Africa; 3 National Health Laboratory Service, Groote Schuur Hospital, South Africa; 4 Division of Clinical Microbiology, University of Cape Town, South Africa; 5 National Institute for Communicable Diseases: Centre for Opportunistic, Tropical and Hospital Infections, National Health Laboratory Service, South Africa; 6 Department of Anatomical Pathology, Red Cross War Memorial Children's Hospital, South Africa; 7 Division of Anatomical Pathology, University of Cape Town, South Africa

## Abstract

Opportunistic fungal infections can cause significant morbidity and mortality in immunocompromised patients. We describe a paediatric case of an unusual disseminated fungal infection. A three-year-old HIV-infected child with severe immunosuppression (CD4+ T-cell count 12 × 10^6^/L) was admitted to hospital with pneumonia, gastroenteritis and herpes gingivostomatitis. Despite antibacterial and antiviral therapy, he experienced high fevers and developed an erythematous maculopapular rash and abdominal tenderness. The child's condition progressively worsened during the admission. A thermally dimorphic fungus was cultured from bone marrow and identified as an Emmonsia species on DNA sequencing. The patient made a good recovery on amphotericin B deoxycholate and antiretroviral therapy. Itraconazole was continued for a minimum of 12 months, allowing for immune reconstitution to occur. This case is the first documented description of disseminated disease caused by a novel Emmonsia species in an HIV-infected child in South Africa.

## Introduction

Opportunistic fungal infections can cause significant morbidity and mortality in immunocompromised patients. The diagnosis and treatment of these infections may be challenging.^1,2^ The manifestation of invasive fungal infection (IFI) is determined by numerous factors including the virulence of the fungal species, the adequacy of host immune responses, the size of the inoculum inhaled or disruption of mucosal barriers. Individuals at risk for IFIs include those who have received transplants, immunosuppressive therapy or chemotherapy for neoplastic diseases; HIV-infected patients; premature infants; and individuals with defects in neutrophil, monocyte, T-lymphocyte or B-lymphocyte function.^3^ In the present report, we describe an unusual disseminated fungal infection caused by a novel Emmonsia species in an HIV-infected child.

## Case report

**FIGURE 1:**
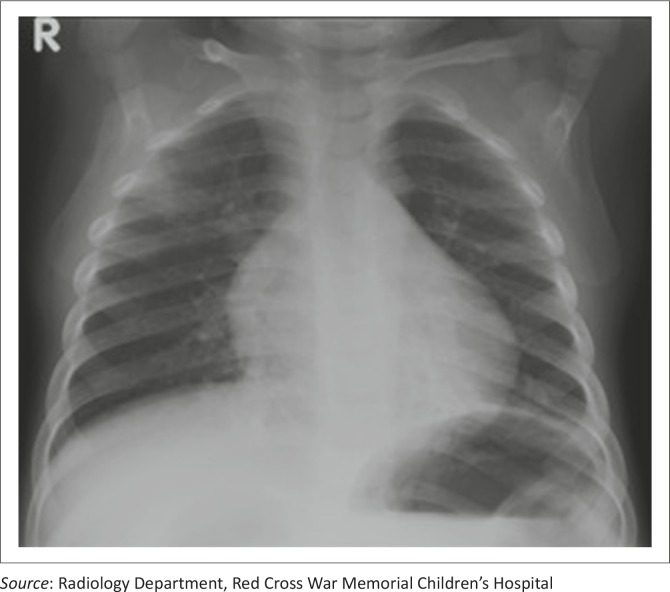
Chest radiograph on admission, with features of bilateral pneumonia.

*Source*: Radiology Department, Red Cross War Memorial Children's Hospital

A 3-year-old boy was admitted to Red Cross War Memorial Children's Hospital, Cape Town, South Africa, with acute gastroenteritis, pneumonia and herpes gingivostomatitis. Six months earlier, HIV infection was diagnosed at a primary health care clinic. Antiretroviral therapy (ART) was not initiated despite it being indicated for such patients by the South African ART guidelines. He was stunted (height 85 cm, height–age z-score – 3) but not wasted (weight 12.6 kg, weight-for-height z-score +1 [2006 WHO Child Growth Standards]). Blood results on admission showed a normocytic anaemia (haemoglobin 6.8 g/dL, reference range 10.7–13.1) and leucopaenia (white cell count [WCC] 2.71 × 10^9^/L, reference range 6–18). The differential WCC showed that neutropaenia (1.22 × 10^9^/L, reference range 2–5.5) and lymphopaenia (1.49 × 10^9^/L, reference range 3.6–12) were present. A chest radiograph showed features of a bilateral pneumonia (Figure 1), for which he was commenced on ampicillin and gentamicin, and acyclovir, for the pneumonia and herpes gingivostomatitis, respectively. No supplemental oxygen therapy was required at admission and empiric treatment for *Pneumocystis* jirovecii pneumonia was not initiated. However, cotrimoxazole prophylaxis was commenced on admission.

He experienced high fevers, peaking at 40 °C. On day 6 of admission, he developed diffuse abdominal tenderness and a generalised erythematous maculopapular exanthem. Liver function tests (LFTs) showed normal total bilirubin (2 µmol/L, ref. range 0–21) and alkaline phosphatase (ALP 268 U/L, ref. range 104–345) concentrations, and elevated γ-glutamyl transferase (GGT 120 U/L, ref. range 3–22), alanine transaminase (ALT 223 U/L, ref. range 5–30) and aspartate transaminase (AST 1304 U/L, ref. range 0–56) concentrations. The C-reactive protein (CRP) was 58.2 mg/L (reference range 0.1–7.5). Owing to the persistent fever, antimicrobial therapy was changed to ertapenem and fluconazole for presumed hospital-acquired infection. There was no growth on blood culture after 5 days of incubation, and a urine culture yielded mixed bacterial growth. Tuberculin skin testing (Mantoux method) and Xpert MTB/RIF (Cepheid, Inc., Sunnyvale, CA, USA) on induced sputum specimens were negative. Two days later, he had generalised tonic clonic seizures for which he received an intravenous loading dose of phenobarbitone. The antibiotic was empirically changed to meropenem for possible hospital-acquired meningitis. A cerebrospinal fluid specimen was bloodstained but there was no bacterial growth, and polymerase chain reaction (PCR) assays for entero- and herpesviruses were negative.

The fever and maculopapular rash persisted. Repeat CRP (88.4 mg/L), procalcitonin (5.3 µg/L, reference range 0.0–0.5), ferritin (91 705 µg/L, reference range 20–200) and triglyceride concentrations (4.7 mmol/L, reference range 0.3–1.1) were elevated. The differential diagnosis included haemophagocytic lymphohistiocytosis (HLH), disseminated tuberculosis and invasive fungal infection. The patient subsequently developed respiratory distress requiring supplemental nasal prong oxygen therapy. Repeat chest radiography was unchanged from admission with features not suggestive of tuberculosis or fungal infection. He was given packed red blood cell, platelet and cryoprecipitate transfusions for severe anaemia (Hb 5.6 g/dL), thrombocytopaenia (platelet count 52 × 10^9^/L) and hypofibrinogenaemia (fibrinogen concentration 1.0 g/L, reference range 1.7 g/L – 4.0 g/L), respectively.

**FIGURE 2:**
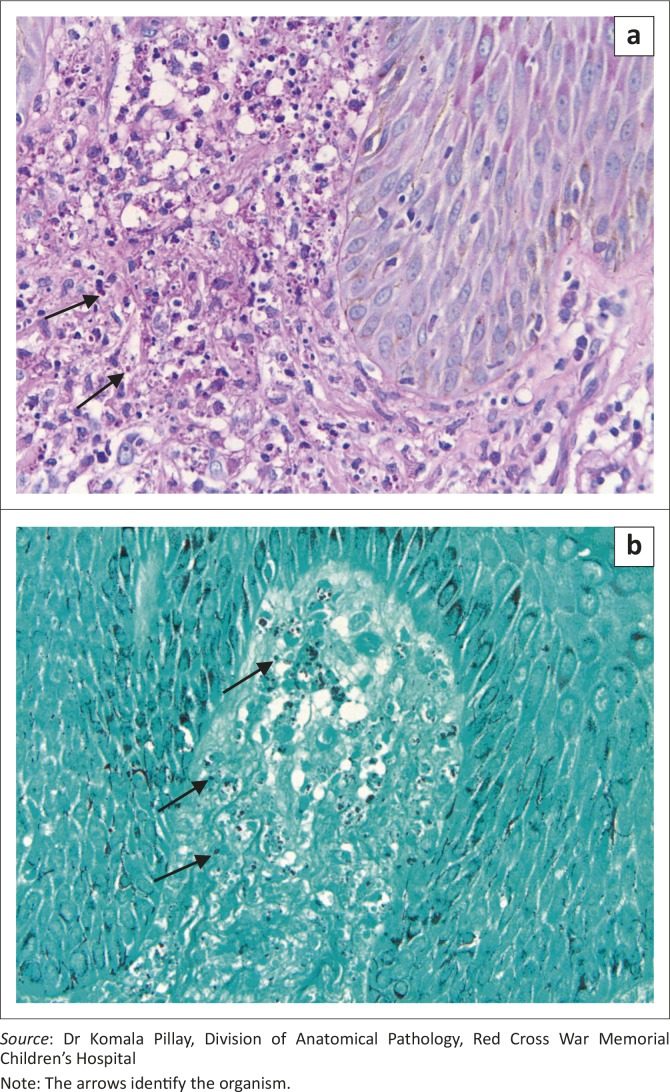
Skin histology biopsy demonstrating fungal elements measuring 1.5 μm** ×** 2.5 μm using (a) Periodic acid-Schiff and (b) Grocott methenamine silver stains.

*Source*: Dr Komala Pillay, Division of Anatomical Pathology, Red Cross War Memorial Children's Hospital

Note: The arrows identify the organism.

Skin and bone marrow biopsies were performed on day 10 in hospital. Skin histology showed extensive karyorrhexis and numerous dermal histiocytes containing cytoplasmic small fungal organisms. Grocott and Periodic acid–Schiff stains were positive and suggestive of dermal histoplasmosis (Figure 2). Skin culture did not grow fungi or myco­bacteria. Bone marrow histology revealed histiocytosis and haemophagocytosis consistent with a diagnosis of HLH. Fungal stains showed scanty structures 1.5 µm × 2.5 µm in diameter and morphologically compatible with *Histoplasma* capsulatum. A diagnosis of disseminated fungal infection with secondary HLH was made and intravenous amphotericin B deoxycholate 1 mg/kg/day commenced 14 days later.

Culture of the bone marrow aspirate in a BACTEC MycoF/lytic bottle (Becton Dickinson, New Jersey) flagged positive after 17.6 days of incubation, and small yeasts were seen on Gram stain. The isolate was submitted to the National Institute for Communicable Diseases for further identification. The fungus was converted from the mycelial to yeast phase on brain heart infusion agar with 5% sheep blood (Diagnostic Media Products, Sandringham) at 35 °C and morphologically characterised based on DNA sequencing of the internal transcribed spacer region of the isolate's ribosomal gene. The isolate was identified as the thermally dimorphic fungus Emmonsia species. The DNA sequence was the same as those of previously described isolates of a novel Emmonsia species. An investigational urine Histoplasma antigen test (ImmunoMycologics, Norman, OK) was negative.

ART, comprising abacavir, lamivudine and efavirenz, was commenced 3 days before amphotericin B was started. The LFT concentrations had marginally improved at this stage with an ALT of 156 U/L, AST 560 U/L and GGT 102 U/L. The baseline CD4+ T-cell count (percentage) was 12 × 10^6^/L (0.45%) and HIV-1 viral load 2 149 305 RNA copies/mL. After ART and amphotericin B were commenced, he made a remarkable recovery. Within 3 days, supplemental oxygen was discontinued and, after 5 days, the skin lesions and fever had resolved. The abdominal tenderness and abnormal LFTs resolved on day 11 and 12 of amphotericin B, respectively.

Daily amphotericin B was continued for 6 weeks via a central venous catheter. Adverse effects were minimised through pre-emptive hydration before amphotericin B infusions, close monitoring of renal function, potassium and magnesium concentrations and, where necessary, electrolyte replacement. Itraconazole 100 mg daily orally was commenced once amphotericin B had been discontinued. Because of its potential for reducing the bioavailability of itraconazole, efavirenz was replaced with a lopinavir/ritonavir co-formulation. Six months after commencing ART, the patient was well, virologically suppressed (HIV-1 viral load < 40 RNA copies/mL), had a CD4+ T-cell (percentage) of 884 × 10^6^ cells/L (17.9%) and normal LFTs. At last evaluation, there was no clinical evidence of active fungal infection. The intention is to maintain him on itraconazole for a minimum of 12 months and until his CD4 count reaches 25%, before considering discontinuation of antifungal therapy.

## Discussion

Emmonsia species, a group of dimorphic fungi, are found in soil, and some species can cause pulmonary infection in rodents.^4^ The genus currently contains three species, namely *Emmonsia crescens*, *Emmonsia parva* and* Emmonsia pasteuriana.* Infection caused by Emmonsia species has been described in HIV-uninfected adults and one child. The infections were caused by *E. crescens* and* E. parva*, resulting in predominantly pulmonary adiaspiromycosis and occasional cutaneous manifestations.^4,5,6,7^ More recently, invasive infection caused by a new species of Emmonsia closely related to *E. pasteuriana* was documented in 13 HIV-infected adults with advanced HIV disease in South Africa.^8^ Our case is the first description of disseminated disease caused by this new Emmonsia species in an HIV-infected child.

All 13 adult patients presented with very low CD4+ T-cell counts, anaemia and widespread skin lesions. The skin manifestations ranged from erythematous papules to plaques and ulcers. Twelve had a documented fever and 8 of 9 who underwent LFTs had deranged liver enzymes, suggesting hepatic involvement. Our patient similarly developed severe immune suppression, anaemia, an erythematous papular eruption and deranged LFTs, and in addition had neutropaenia and lymphopaenia. In that series, the original histological diagnosis on skin biopsy samples morphologically resembled *H. capsulatum.* On gene sequencing, the fungus was identified as belonging to the genus Emmonsia.^8^ Not all fungi require molecular diagnosis. However, Emmonsia species is difficult to diagnose by morphological appearance alone, necessitating molecular confirmation. The role of the Beta D Glucan assay in the diagnosis of emmonsiosis is currently not known. The assay is likely to be elevated but, as it detects most common fungi, is unlikely to be specific for Emmonsia species.

Our patient was empirically commenced on fluconazole during the course of his clinical deterioration. Fluconazole is less active than itraconazole against dimorphic fungi especially histoplasmosis, probably explaining the lack of clinical response until amphotericin B deoxycholate was commenced.^9,10^ Currently there are no treatment guidelines for HIV-associated disseminated Emmonsia infection. Our regimen of amphotericin B deoxycholate followed by maintenance itraconazole therapy was extrapolated from treatment guidelines for other dimorphic fungal infections.^11^ Duration of therapy depends on the extent of the disease. As our patient had disseminated fungal infection with severe immunosuppression, we elected to follow recommendations for treating disseminated histoplasmosis in HIV-infected patients. Lifelong antifungal prophylaxis may not be required in our patient because he has already had a good immunological response.^12^

Itraconazole is metabolised by the cytochrome P450 C3A4 enzyme. This pathway is also responsible for the metabolism of the non-nucleoside reverse transcriptase (NNRTI) class of antiretroviral drugs. Theoretically, levels of either the itraconazole or NNRTI could be affected. There is a paucity of literature describing the drug–drug interactions between itraconazole and the NNRTIs. A case report of an HIV-infected male patient with disseminated Histoplasma infection demonstrated reduced levels of itraconazole and an increase in the urine Histoplasma antigen level while he was receiving efavirenz. After switching to a protease inhibitor, the serum drug levels of itraconazole increased and the urine Histoplasma antigen levels declined.^13^ Drug levels of itraconazole were not monitored in the case described because the test is not available in South Africa.

## Conclusion

The recently published case series and the present case report confirm that emmonsiosis occurs among both HIV-infected adults and children in South Africa.^8^ Prolonged incubation (up to 6 weeks) of the fungal culture may be necessary for the diagnosis. This infection should be considered in immunocompromised patients presenting with persistent fever and other clinical features suggestive of IFI.
